# Evaluation of Stability, Inactivation, and Disinfection Effectiveness of Mpox Virus

**DOI:** 10.3390/v16010104

**Published:** 2024-01-11

**Authors:** Yuwei Li, Shiyun Lv, Yan Zeng, Zhuo Chen, Fei Xia, Hao Zhang, Demiao Dan, Chunxia Hu, Yi Tang, Qiao Yang, Yaqi Ji, Jia Lu, Zejun Wang

**Affiliations:** 1 Wuhan Institute of Biological Products Co., Ltd., Wuhan 430200, China; wibpliyuwei@126.com (Y.L.); lvshiyun180221@163.com (S.L.); wyz140220@163.com (Y.Z.); chenzhuo_zz@163.com (Z.C.); 19907110481@163.com (F.X.); 2015301060083@whu.edu.cn (H.Z.); ddmiao022@163.com (D.D.); huchunxiawh@126.com (C.H.); 2019202040121@whu.edu.cn (Y.T.); yangqiao-s@163.com (Q.Y.); jyq0718@163.com (Y.J.); 2 State Key Laboratory for Novel Vaccines Research and Development of Emerging Infectious Diseases, Wuhan 430200, China

**Keywords:** MPXV, stability, heat, BPL, inactivation, disinfection

## Abstract

Background: Mpox virus (MPXV) infections have increased in many countries since May 2022, increasing demand for diagnostic tests and research on the virus. To ensure personnel safety, appropriate and reliable measures are needed to disinfect and inactivate infectious samples; Methods: We evaluated the stability of infectious MPXV cultures stored at different temperatures and through freeze–thaw cycles. Heat physical treatment (56 °C, 70 °C, 95 °C), chemical treatment (beta-propiolactone (BPL)) and two commercialized disinfectants (Micro-Chem Plus (MCP) and ethanol) were tested against infectious MPXV cultures; Results: The results indicated that MPXV stability increases with lower temperatures. The MPXV titer was stable within three freeze–thaw cycles and only decreased by 1.04 log_10_ (lg) 50% cell culture infective dose (CCID_50_) per milliliter (12.44%) after twelve cycles. MPXV could be effectively inactivated at 56 °C for 40 min, 70 °C for 10 min, and 95 °C for 5 min. For BPL inactivation, a 1:1000 volume ratio (BPL:virus) could also effectively inactivate MPXV. A total of 2% or 5% MCP and 75% ethanol treated with MPXV for at least 1 min could reduce >4.25 lg; Conclusions: MPXV shows high stability to temperature and freeze–thaw. Heat and BPL treatments are effective for the inactivation of MPXV, while MCP and ethanol are effective for disinfection, which could help laboratory staff operate the MPXV under safer conditions and improve operational protocols.

## 1. Introduction

The mpox virus (MPXV) was first isolated from laboratory monkeys suffering from a pox-like disease in Copenhagen, Denmark, in 1958 [1,2]. The first human case reported was in 1970 in a child in the Democratic Republic of Congo (DRC) [3]. MPXV is a member of the Orthopoxvirus family of Poxviridae. MPXV is a double-stranded DNA virus of the genus orthopoxviruses, which also includes variola, cowpox (CPX), and vaccinia viruses. MPXV has emerged into two distinct clades, clade I (formally the Congo Basin or Central African clade) and clade II (formally the West African clade) [4]. Compared to clade II, clade I caused a disease with a higher fatality rate [5]. MPXV causes infection with a clinical presentation resembling smallpox (SPX), which is caused by the variola virus (VARV) infection. Human mpox is a zoonotic disease that was confined to Central and Western Africa’s forested areas in the twentieth century [6]. Since early May 2022, cases of mpox have been reported in countries where the disease is not endemic and continue to be reported in several endemic countries [7]. Globally, the outbreak continues to spread across regions and populations [8].

On 11 May 2023, the World Health Organization (WHO) declared that the multi-country outbreak of mpox was no longer a public health emergency of international concern [9]. Nevertheless, mpox prevention and control is still necessary. According to the WHO, fourteen countries reported increased cases in the last three weeks (21 August through 10 September 2023) compared to the three weeks prior (31 July through 20 August 2023) [10]. In addition, indirect transmission of infection cases in hospitals has been reported [11]. This suggests that it may be due to the fact that particles released by infected people can deposit on environmental surfaces and can survive for hours to days, resulting in transmission through indirect contact with contaminated surfaces. Therefore, a crucial preventive measure against mpox is the sufficient inactivation of MPXV using available disinfection methods. In addition, inactivated viruses were also needed in clinical diagnosis and many research laboratories for further study. For laboratory biosafety, specimens delivered from high-level biocontainment areas should be inactivated properly and reliably.

It has been reported that MPXV virions (clade II) could be recovered from stainless steel discs after several days, including up to 30 days when stored at 4 °C [12], suggesting that contamination in laboratories and surfaces frequently touched by objects in the patient’s living environment is a potential source of virus transmission, and it can be assumed that the virus would remain infectious for longer periods in liquid environments. Nevertheless, there are limited data on mpox virus stability and inactivation under various environmental conditions, and vaccinia viruses were usually used for simulation evaluations [13]. In this study, the effects of storage conditions and heat treatment on viral activity were evaluated, as well as the virucidal activity of beta-propiolactone (BPL) exposure and commercially available surface disinfectants. Additionally, MPXV could pose a risk to those handling clinical samples in basic research and healthcare. The results of this study provide useful information for securing the safety of handling infectious MPXV specimens.

## 2. Materials and Methods

### 2.1. Cells and Virus

Vero E6 cells were cultured at 37 °C with 5% CO_2_ in Dulbecco’s Modified Eagle’s medium (DMEM, Gibco, Waltham, MA, USA) supplemented with 10% newborn bovine serum (NBS, Hangzhou, China) and 50 units/mL penicillin–streptomycin (Gibco, Waltham, MA, USA). MPXV (WIBP-MPXV-001) belonging to clade II was isolated from clinical specimens by Biosafety (level 3) Laboratory, Wuhan Institute of Biological Products Co., Ltd. MPXV was propagated in Vero E6 cells in DMEM supplemented with 2% NBS, and 50 units/mL penicillin–streptomycin. All studies of infectious mpox virus were performed in the biosafety level 3 facility in Wuhan.

### 2.2. Virus Culture and Titration

The Vero E6 monolayer grown in a T225 flask was infected with MPXV at 0.5 multiplicity of infection (MOI). After that, cells were incubated at 37 °C with 5% CO_2_ for four days and analyzed under a microscope. After discarding the supernatant, the cells were washed with phosphate-buffered saline (PBS, Sango Biotech, Shanghai, China), and lysed by performing 3 freeze–thaw cycles at −80 °C. Then, the supernatants were collected by centrifuging at 3000 rpm for 5 min. To determine the virus concentration, a 10-fold serially diluted stock virus (supernatant) was added to the 96-well plate with 2~3 × 10^4^ Vero cells per well prepared one day before. Cells were examined under a microscope for cytopathological effects after 5 days of culture at 37 °C, 5% CO_2_. Virus titer was determined by the method of Karber to determine the 50% cell culture infective dose (CCID_50_) [14,15].

### 2.3. Temperature and Freeze–Thaw Cycle Stability

Aliquots of MPXV cultures were stored separately at 37 °C, room temperature (20~22 °C, RT), and 4 °C. The MPXV aliquots were titrated every seven days according to Karber’s method. Furthermore, samples were frozen at −80 °C and thawed at room temperature to evaluate the effects of 3, 6, 9, and 12 freeze–thaw cycles. The fully thawed aliquots were titrated according to Karber’s method.

### 2.4. Heat Inactivation

MPXV cultures were thawed at room temperature. Prepare aliquots of each concentration (0.5 mL) in 2 mL screw-capped tubes (Axygen, Tewksbury, MA, USA). Aliquots were heated at 56 °C, 70 °C, or 95 °C for different times, ranging from 2 min to 50 min using a dry thermostat. After heating, aliquots were titrated using Karber’s method and verified for inactivation. The titer was given a value of 1 when no cytopathic effects (CPE) was observed.

### 2.5. BPL Inactivation

MPXV cultures (7.13 lg CCID_50_/mL) were added to a T25 flask and treated with beta-propiolactone (BPL) (SERVA Electrophoresis GmbH, Heidelberg, Germany) at final volume ratio of 1:1000 or 1:2000. The mixture was incubated at 2–8 °C for 24 h and then hydrolyzed at 37 °C for 4 h. Control supernatants were treated similarly to BPL-treated samples. In control experiments, BPL was tested for cell toxicity.

### 2.6. Validation of the Inactivation

Vero cells were incubated with treated samples for three consecutive generations as follows: Inactivated MPXV cultures were inoculated into Vero monolayers in 12-well plates. The first passage cells were cultured at 37 °C with 5% CO_2_ for 4 days. After freeze–thaw cycles, the cell mixture was inoculated onto new Vero monolayers in 12-well plates at 37 °C with 5% CO_2_ for 4 days. After that, the third passage was performed according to the second passage’s method. Afterward, MPXV with MOI 0.01 was added to the cultured cells at 37 °C with 5% CO_2_ for 4 days for cell infection. For each generation, cells were evaluated for morphological changes. If CPE was observed under the microscope, it was marked as “+”, and no CPE was marked as “−”. No CPE was observed for three generations, and CPE was observed after infection to prove successful inactivation. Y represented completely inactivated samples (no CPE), and N represented samples whose inactivation failed. Inactivation verification was used to evaluate the effects of heat inactivation and BPL inactivation.

### 2.7. Virucidal Activity of Disinfectants

The common decontamination reagents as the quaternary ammonium salt disinfectant Micro-Chem Plus™ Detergent Disinfectant (MCP, National Chemical Laboratories, Philadelphia, PA, USA) and the alcohol disinfectant 75% ethanol (MDS, Wuhan, China) were tested for viral disinfection. An inactivation effect of chemical disinfectants against MPXV was evaluated using a quantitative suspension method. Firstly, the disinfectant and neutralizing agent mixtures were added to Vero E6 cells separately and incubated for cytotoxicity assessment. Dilute 0.1 mL of organic interferents 2-fold, mixed with 0.8 mL of disinfectants, and then add the neutralizing agent (a mixture of 10 g/L sodium thiosulfate, 1% Tween-80, and 10 g/L lecithin as a neutralizing agent for 2% or 5% MCP, 3% polysorbate 80 as the neutralizer of 75% ethanol). After a 10-fold dilution of the mixture, 0.1 mL was added to Vero E6 cells and incubated at 37 °C with 5% CO_2_ for 1 h. The dilution was discarded, and cells were covered with fresh medium. Cells were examined under a microscope for CPE after 5 days of culture at 37 °C, 5% CO_2_. After ensuring that there is no cytotoxicity, the inactivation effect of chemical disinfectants against MPXV was evaluated using a quantitative suspension method. An amount of 0.1 mL of organic interference material was mixed with 0.1 mL MPXV at RT for 5 min, and 0.8 mL of disinfectant was added and mixed. Then the neutralizer was added immediately and diluted 10-fold after 30, 1, and 5 min to terminate the disinfection reaction. The mixture titration was quantified using Karber’s method.

### 2.8. Statistical Analysis

GraphPad Prism 8 software (GraphPad Software, San Diego, CA, USA) was used for all statistical analyses. *t*-tests were used to analyze the data between two groups. *p* < 0.05 was considered statistically significant. Not significant (ns), *p* > 0.05; * *p* < 0.05; ** *p* < 0.01; *** *p* < 0.001; **** *p* < 0.0001.

## 3. Results

### 3.1. Temperature Stability

**Figure 1:**
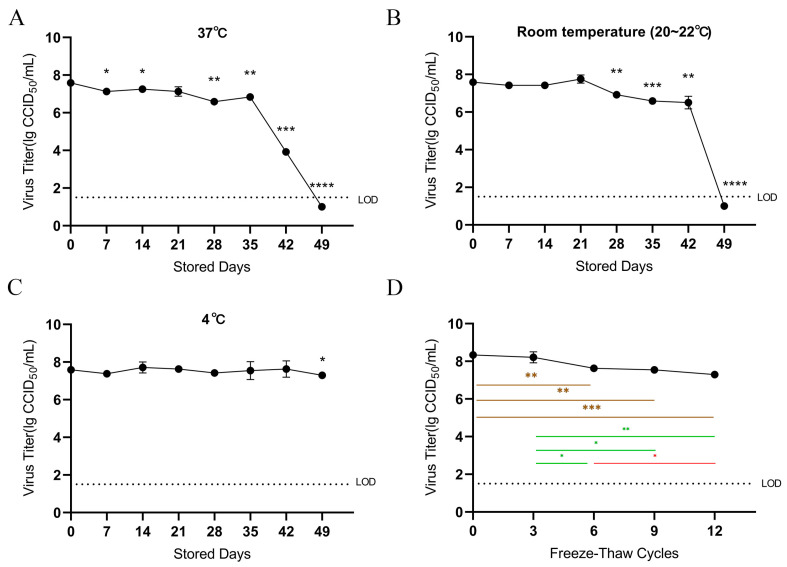
The stability of temperature and freeze–thaw cycles. Stability of mpox virus (MPXV) at 37 °C (**A**), room temperature (20~22 °C, (**B**)), or 4 °C (**C**). The MPXV aliquots stored at 37 °C (**A**), room temperature (20~22 °C, (**B**)), or 4 °C (**C**) were titrated every seven days according to Karber’s (Ramakrishnan, 2016) method. Results are expressed as the mean±standard deviation of 3 independent experiments (*n* = 3). Limit of detection (LOD), 1.5 log_10_ (lg) 50% cell culture infective dose (CCID_50_) per milliliter. (**D**) The virus titer stability after repetitive freeze–thaw cycles of three samples. The curve values represent means ± SD (*n* = 3/group), significant differences between repetitive freeze–thaw cycles and negative control are shown. *t*-test analyses were used to analyze the data. *p* < 0.05 was considered statistically significant. Not significant (ns), *p* > 0.05; * *p* < 0.05; ** *p* < 0.01; *** *p* < 0.001; **** *p* < 0.0001.

In this study, we evaluated the stability of MPXV. Aliquots of MPXV cultures were stored separately at 37 °C, room temperature (RT, 20~22 °C), and 4 °C for 49 days. The initial virus titer was 7.5 lg CCID_50_/mL. After being stored at 37 °C for 7 days, the virus titer was slightly decreased (Figure 1A). However, the virus titer still maintained 90.15% (6.84 lg CCID_50_/mL) of the initial titer on the 35th day. The virus titer kept constant for the first 21 days at 20~22 °C, 42 days at 4 °C (Figure 1B,C), and then started to decrease. Similarly, the virus titer still reached 85.76% (6.50 lg CCID_50_/mL) of the original titer on the 42nd day of storage at RT. At 49 days, the virus titer of samples stored at 4 °C showed only a slight downward trend, while 37 °C and RT samples were below the detection limit. Overall, the stability of MPXV is strongly influenced by temperature, and the higher the temperature, the more likely the virus is to be inactivated.

### 3.2. Freeze–Thaw Cycle Stability

Freeze–thaw tests were performed to determine whether MPXV titer is affected by freeze–thawing. There is no significant difference between the three freeze–thaw cycles and the fresh samples (*p* = 0.5547). However, the titer decreased by 0.70 lg CCID_50_/mL (8.44%) after six freeze–thaw cycles, significantly lower than fresh samples (*p* = 0.003). After nine cycles, the virus titer was significantly decreased by 0.79 lg CCID_50_/mL (9.44%) compared to the initial samples (*p* = 0.0045), while there was not detectably a difference from the titer after six cycles (*p* = 0.5585). In addition, the virus titer dropped by 1.04 lg CCID_50_/mL (12.44%) after twelve freeze–thaw cycles (*p* = 0.0004) and was significantly lower than the six freeze–thaw cycles (*p* = 0.0167), while there was no significant difference after nine times of freeze–thaw (*p* = 0.1000).

### 3.3. Heat Inactivation

MPXV cultures with a titer of 7.13 lg CCID_50_/mL were used for heat inactivation validation. The data showed that when the temperature was 56 °C, after heating for 5 min, the virus titer decreased to 5.97 ± 0.28 lg CCID_50_/mL (mean ± SD); after 10 min, the virus titer decreased to 3.64 ± 0.9 lg CCID_50_/mL; after 20 min, the virus titer decreased to 1.88 lg CCID_50_/mL; and after heating for 40 min, the virus could be completely inactivated (Table 1A). When the temperature was increased to 70 °C, after heating for 2 min, the virus titer decreased to 6.38 ± 0.05 lg CCID_50_/mL. After heating for 5 min, the virus titer decreased below the detection limit, however, CPE was found in the cells during the third passage through inactivation verification, which indicated that the virus inactivation failed. After heating for 10 min, the virus could be completely inactivated (Table 1B). When the temperature was increased to 95 °C, after heating for 2 min, the virus titer decreased to 1.63 ± 0 lg CCID_50_/mL. Inactivation of MPXV requires only 5 min (Table 1C).

### 3.4. BPL Inactivation Method

MPXV cultures with titer 7.13 lg CCID_50_/mL were incubated with BPL at final concentrations of 1:1000 or 1:2000 at 4 °C for 24 h. According to the results of three generations of analysis, CPE was observed in samples inactivated with 1:2000 BPL and no CPE was observed in samples inactivated with 1:1000 BPL. At the same time, no cytotoxicity caused by BPL of this concentration was observed in controlled experiments (Table 2).

### 3.5. Virucidal Activity of Disinfectants

A culture of MPXV was disinfected with 75% ethanol, 2% or 5% MCP. All sterilized virus titers were below the limit of detection, and the unsterilized virus control titer was 5.75 lg CCID_50_/mL. The results showed that virus titers could be reduced by at least 4.25 lg CCID_50_/mL after mixing 75% ethanol, 2% or 5% MCP for 1 min or 5 min (Table 3).

## 4. Discussion

In countries with the historical transmission of mpox and their neighboring countries, the WHO has assessed the risk as moderate [10]. On 10 May 2023, the WHO Director-General determined that the mpox outbreak no longer constituted a public health emergency of international concern [9]. Many countries have experienced an increase in human mpox virus infections. The virus can be transmitted to humans by contact with infected people, contaminated materials, or animals infected with it [16]. All of this information reminds us of the need for continued research into the characteristics of the mpox virus to enable more comprehensive public health and laboratory safety disinfection and inactivation measures to prevent possible future outbreaks. Enveloped DNA viruses, e.g., poxviruses and hepatitis B virus, are more sensitive to detergents and disinfectants than non-enveloped RNA viruses, e.g., enteroviruses and human parechoviruses [17,18,19,20]. It is well known that poxvirus virions have long-term persistence in the environment [21,22]. Currently, there is little information about inactivating against mpox specifically. Actually, the majority of publications, from life cycle to environmental persistence, were based on research with vaccinia virus and smallpox, which belong to the same orthopoxvirus genus as MPXV [17,23,24]. It has been reported that vaccinia and smallpox have a strong environmental persistence on surfaces and in solution, such as smears from patients, VACV M1-spiked stormwater, and variola virus in scabs [25,26,27].

According to stability studies, the titer of MPXV stored at 37 °C and 20–22 °C for up to 35 days could maintain more than 86.86% activity. The findings were consistent with recent reports in which infectious MPXV survives in the home for more than 15 days in an ambient temperature environment [28]. In addition, the virus could be stored at 4 °C for more than 49 days. As a result, virus stability increases at lower temperatures. For freeze–thaw cycles, we evaluated the effects of three, six, nine, and twelve freeze–thaw cycles on the virus titer, and the results showed that the virus titer did not decrease significantly for every three freeze–thaw cycles, but would decrease significantly if it continued to increase to six times of freeze–thaw. Although the titer of the virus decreased due to freeze–thaw, compared with the original virus titer of 8.33 lg CCID_50_/mL, the virus was still as high as 7.29 lg CCID_50_/mL after 12 freeze–thaw cycles. Alex W H Chin et al. reported that SARS-CoV-2 can persist for 14 days in Dulbecco’s Modified Eagle medium at 4 °C whereas the persistence time was dramatically reduced to 10 min and 1 min when the temperature was increased to 56 °C and 70 °C, respectively [29]. Manman Dai et al. reported that SARS-CoV-2 in culture medium remained infectious at 4 °C and 25 °C for more than 8 days, and the untreated virus titer was more stable at 4 °C than 25 °C [30]. This indicates that the stability of the mpox virus was higher than that of COVID-19, which means that laboratories conducting MPXV experimental activities, clinical medical staff, and the general public have higher requirements for the correct selection of inactivation conditions and disinfectants in the prevention of MPXV.

A culture solution of MPXV was heated at different temperatures for various durations. Data showed MPXV was inactivated at 56 °C for more than 40 min, 70 °C for more than 10 min, and 95 °C for more than 5 min. This was consistent with the conditions recommended in many guidelines. This effective inactivation condition requires higher requirements, compared to reports that MPXV can effectively inactivate at 56 °C for 10 min (titer reduction > 6.9 lg TCID_50_/mL) [31]. Some studies have also shown that MPXV can be inactivated at 70 °C for less than 5 min, which is less than the required 10 min in this study [32]. This may be due to the fact that this study used three consecutive passages for inactivation verification, which is more stringent and safer than the general experiment of only observing CPE effects through one passage culture to determine whether the virus is inactivated. Virus inactivation was also effective with the 70 °C temperature recommended in some extraction kits. However, the virus titer selected in this experiment was 7.13 lg CCID_50_/mL and the sample volume was 0.5 mL. It may be necessary to increase the inactivation temperature or extend the inactivation time if the virus’s titer or volume exceeds these values.

There have been reports of BPL inactivating viruses such as SARS-CoV-2 successfully, and BPL has been approved by regulatory agencies in various countries for the production of virus-inactivated biological products [33,34,35,36]. At the same time, BPL is easily hydrolyzed into non-toxic beta-hydroxy propionic acid at 37 °C for several hours and will not affect the subsequent experiment [37]. When treated with BPL, viruses retain their antigenicity and nucleic acid stability [38,39,40]. With regard to BPL treatment, the results indicate that low concentrations of BPL (final volume concentrations 1:2000) failed to inactivate MPXV after 24 h. However, higher concentrations of BPL (final concentrations 1:1000) led to the virus being inactivated within 24 h. The inactivation method is suitable for experiments where the integrity of nucleic acids and natural antigens is critical.

As a commonly used disinfectant in biosafety laboratories, 5% MCP and 75% ethanol only need 1 min to reduce the titer of the mpox virus by at least 4.25 lg CCID_50_/mL. The WHO guidelines for robust and reliable viral safety include the requirement that virucidal substances can remove or inactivate at least 4 lg of virus as a standard acceptance criterion [41,42]. Therefore, 5% or 2% MCP and 75% ethanol can be used for sanitizing in clinical and laboratory settings. It has been reported that quantitative suspension experiments with 70% ethanol (≤1 min) showed that different vaccinia virus strains could be inactivated by at least 4 lg, which is consistent with our research results on the mpox virus [13,43,44,45,46]. G. Kampf suggested that it is reasonable to assume that different types of smallpox viruses have similar susceptibility to disinfectants, which was also confirmed by our results [13].

In fact, WHO, U.S., European, and other national Centers for Disease Control and Prevention have issued prevention and control recommendations for MPXV [47]. In this study, we developed MPXV inactivation procedures that can safely be applied to a variety of specimen types and research purposes. The findings should assist medical institutions and laboratories in developing or improving standard inactivation methods.

We established MPXV inactivation procedures that can safely be applied to a variety of specimen types and research purposes. Considering the different centrifuge tubes selected by different laboratories, the titer of the virus to be inactivated is different, and the time required for the virus to heat to the target temperature may be considerably longer. In practice, the thermal inactivation time should be prolonged as much as possible. If the required inactivated virus titer is higher than the virus titer in this study, it should be re-validated. At the same time, when selecting the inactivation method, we should consider whether it will affect the structural integrity of the virus, which is related to whether it will affect experimental results.

Overall, our research has demonstrated that MPXV has high stability to temperature and freeze–thaw, but effective virus inactivation of MPXV replication can be achieved through heat, BPL inactivation methods, and commercial disinfectants used for virus disinfection. To conduct MPXV research at lower biocontainment levels, 65 °C, 70 °C, or 95 °C for more than 40 min, 10 min, and 5 min, respectively, and BPL (final volume concentration 1:1000) at 4 °C for 24 h could be effective methods of inactivation. The findings of this study should be used to improve and approve standard operating procedures for inactivation, which could ensure greater safety for personnel and the environment. However, there are still some limitations to this study. Medical institutions may be exposed to more types of environments and specific body fluids, such as blood, saliva, urine, feces, semen, skin, rectal and oropharyngeal swab specimens, which have been detected in various samples [48,49]. Yinda et al. have evaluated the stability of MPXV in specific body fluids and found that the half-life of the virus in the wet and dry phases of blood, semen, and serum was similar to that in DMEM solution [50]. Moreover, the environmental inactivation of MPXV can be slowed by proteins in the solution [50]. Therefore, we can reasonably speculate that the results of this study can be used as a reference for the development of inactivation or disinfection procedures in healthcare institutions and laboratories. However, in order to ensure the safety of people and the environment, further safety testing is required if disinfection or inactivation conditions are changed.

## Figures and Tables

**Table 1:** (A) Heat inactivation at 56 °C. (B) Heat inactivation at 70 °C. (C) Heat inactivation at 95 °C. ND: not detected (below the limit of virus detection: 1.5 log_10_ (lg) 50% cell culture infective dose (CCID_50_) per milliliter). CPE: cytopathic effects.

Temp	Time	Virus Titer (lg CCID_50_/mL; Mean ± SD)		CPE (1/2/3 Passage)	Inactivated (Y/N)
		Pre-Heating	After-Heating		
(A)					
56 °C	5 min	7.13	5.97 ± 0.28	+/+/+	N
	10 min		3.64 ± 0.9	+/+/+	N
	20 min		1.88 ± 0	−/+/+	N
	30 min		ND	−/+/+	N
	40 min		ND	−/−/−	Y
	50 min		ND	−/−/−	Y
(B)					
70 °C	2 min	7.13	6.38 ± 0.05	+/+/+	N
	5 min		ND	−/−/+	N
	10 min		ND	−/−/−	Y
	15 min		ND	−/−/−	Y
	20 min		ND	−/−/−	Y
	30 min		ND	−/−/−	Y
(C)					
95 °C	2 min	7.13	1.63 ± 0	−/+/+	N
	5 min		ND	−/−/−	Y
	10 min		ND	−/−/−	Y
	15 min		ND	−/−/−	Y
	20 min		ND	−/−/−	Y
	30 min		ND	−/−/−	Y

**Table 2:** beta-propiolactone (BPL) chemical inactivation.

BPL:Virus	Time (h)	CPE (1/2/3 Passage)	Inactivated (Y/N)
1:1000	24	−/−/−	Y
1:2000		−/+/+	N

**Table 3:** Chemicals tested for viral disinfection. Reduction factors (lg) of three disinfectants against mpox virus (MPXV). ND: not detected (below the limit of virus detection: 1.5 lg CCID_50_/mL). MCP: Micro-Chem Plus^TM^.

Chemicals	Concentration (*v*/*v*)	Time (min)	Virus Control Titer (Undisinfected; lg CCID_50_/mL)	Virus Titer (Disinfected; lg CCID_50_/mL)	Reduction Factor (lg)
MCP	5%	1	5.75	ND	≥4.25
		5		ND	≥4.25
	2%	1		ND	≥4.25
		5		ND	≥4.25
Ethanol	75%	1		ND	≥4.25
		5		ND	≥4.25

## Data Availability

Data are contained within the article.
